# Mitochondrial genome characterization and phylogeny of *Wallacea dactyliferae* Maulik 1919 (Coleoptera: Chrysomelidae) from China

**DOI:** 10.1080/23802359.2022.2151830

**Published:** 2022-12-07

**Authors:** Song Yang, Guangxun Yang, Zhongping Xiong, Jian Huang

**Affiliations:** a Yunnan Academy of Biodiversity, Southwest Forestry University, Kunming, China; b Yunnan General Administration of Forestry Seeds and Seedlings, Kunming, China

**Keywords:** Mitochondrial genome, Wallacea dactyliferae, Palm pest, Coleoptera, Chrysomelidae, Cassidinae

## Abstract

*Wallacea dactyliferae* Maulik 1919 (Coleoptera: Chrysomelidae) has been reported as a new invasive palm pest in Asia recently. So far, a total of 29 species have been reported in *Wallacea*. In the present study, the whole mitochondrial genome of *W. dactyliferae* was identified for the first time (also for the first species of *Wallacea*) by using high throughput sequencing systems. The entire genome is 16,243 bp in length (ACCN: OK513040) consisting of 13 protein-coding genes, 2 ribosomal RNA genes, 22 transfer RNA genes, and an A + T-rich region. Phylogenetic analysis revealed that insects from the same subfamily were clustered together, with *W. dactyliferae* being clustered together with other Cassidinae species. This study can provide essential DNA molecular data for further phylogenetic and evolutionary analyses for Chrysomelidae family of the Coleoptera order.

So far, a total of 29 species have been reported in *Wallacea* (Coleoptera: Chrysomelidae) (Sekerka 2015, Lee and Sekerka 2018), all distributed in the Oriental Region. *Wallacea dactyliferae* was first reported to feed on date palm *Phoenix dactylifera* in India (Maulik 1919) and has become a serious pest on *P. canariensis*, *Chamaerops humilis* and *Washingtonia filifera* in France (Drescher and Martinez 2005) following accidental introduction. *Wallacea dactyliferae* has been reported as a new invasive palm pest in Asia recently. It originated in southern India and then spread to Bangladesh, Vietnam, Thailand, Taiwan and Yunnan of China (Sekerka 2015; Lee and Sekerka 2018; Wu 2019). This pest has a certain ability to invade a new environment, and may further spread the danger in China in the future.

In this study, *W. dactyliferae* was collected in 2021 in Anning (Latitude: 24.9461 N; Longitude: 102.4914E) of Yunnan province, China and the specimen (voucher no. M2021-0127; Figure 1) was deposited in the Insect Systematics and Diversity Lab at Yunnan Academy of Biodiversity, Southwest Forestry University, Kunming, China (https://yab.swfu.edu.cn/; contact person, Song Yang, email: yangsong@swfu.edu.cn). Genomic DNA was extracted from the whole body of a single *W. dactyliferae* pupa using the CTAB method (Doyle and Doyle 1987). The isolated DNA was sheared to 300-500 bp fragments in a Covaris (KBiosciences) ultrasonicator device. The sequencing cDNA libraries were prepared by using TruSeq Nano DNA Sample Prep Kit (Illumina, San Diego, CA) following the manufacturer’s protocols. The library preparations were sequenced on an Illumina NovaSeq 6000 platform and 150 bp paired-end reads were generated. The clean reads were generated from the raw reads by trimming adapters and low quality reads by using Trimmomatic v0.39 (http://www.usadellab.org/cms/index.php?page=trimmomatic). The mitochondrial genome of *W. dactyliferae* was assembled by using NOVOPlasty (Dierckxsens et al. 2017) and genes within the genome were predicted by DOGMA (Wyman et al. 2004) and MITOS (Bernt et al. 2013).

**Figure 1.**
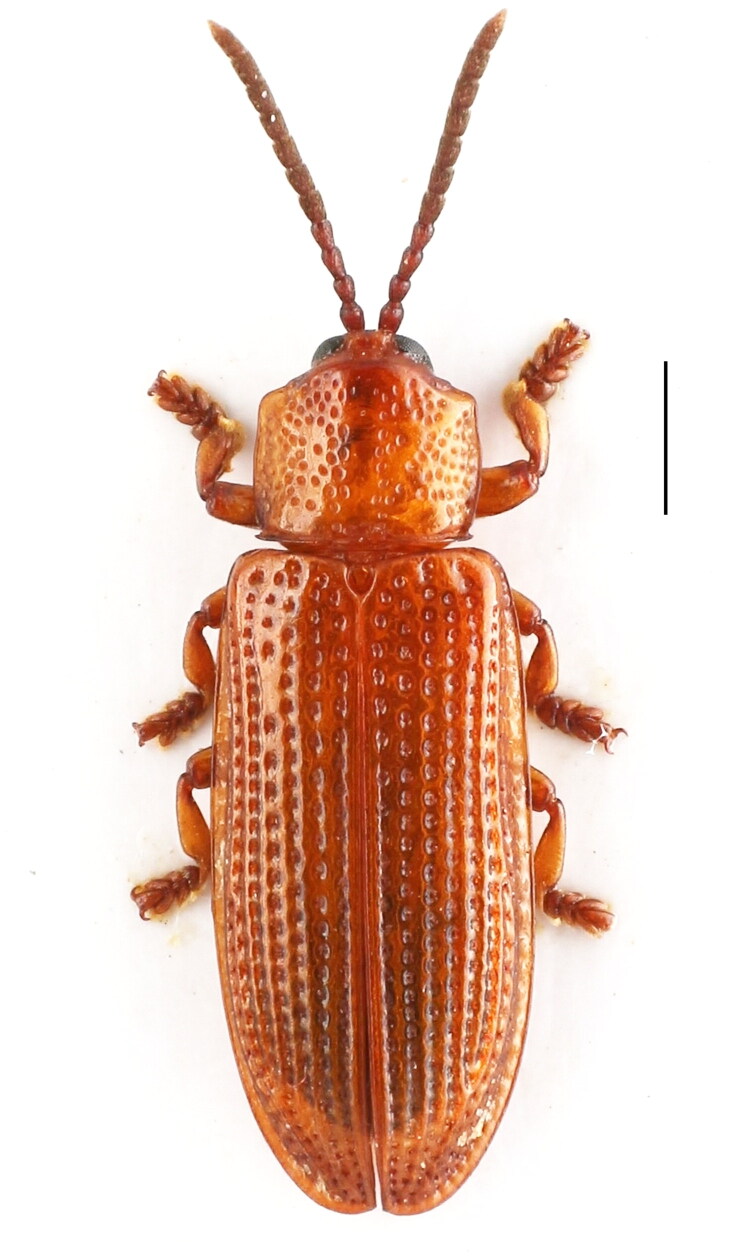
*Wallacea dactyliferae*. Scale = 1 mm. This is an original image by the authors.

The *W. dactyliferae* mitochondrial genome (GenBank ID: OK513040) was 16,243 bp in size, circular and double-stranded, with GC content being 23.01% (Figure 2). This genome has 2 rRNAs, 22 tRNAs and 13 protein-coding genes (PCGs), and a non-coding A + T-rich region (1696 bp). The minority strand (N-strand) of the genome encodes 14 genes, including 2 rRNAs, 8 tRNAs and 4 PCGs; and the majority strand (J-strand) encodes 23 genes, including 14 tRNAs and 9 PCGs. Three kinds of start codons are found in PCGs, namely, ATT, ATA and ATG. Five PCGs end with TAA and one PCG ends with TAG, and the other PCGs have incomplete stop codons, with one being TA- and six being T–. The order and orientation of the above mitochondrial genes, as well as the start and end codons used by these genes were similar to other Cassidinae insects (Xu et al. 2018; Yan et al. 2021; Zhang et al. 2021).

**Figure 2.**
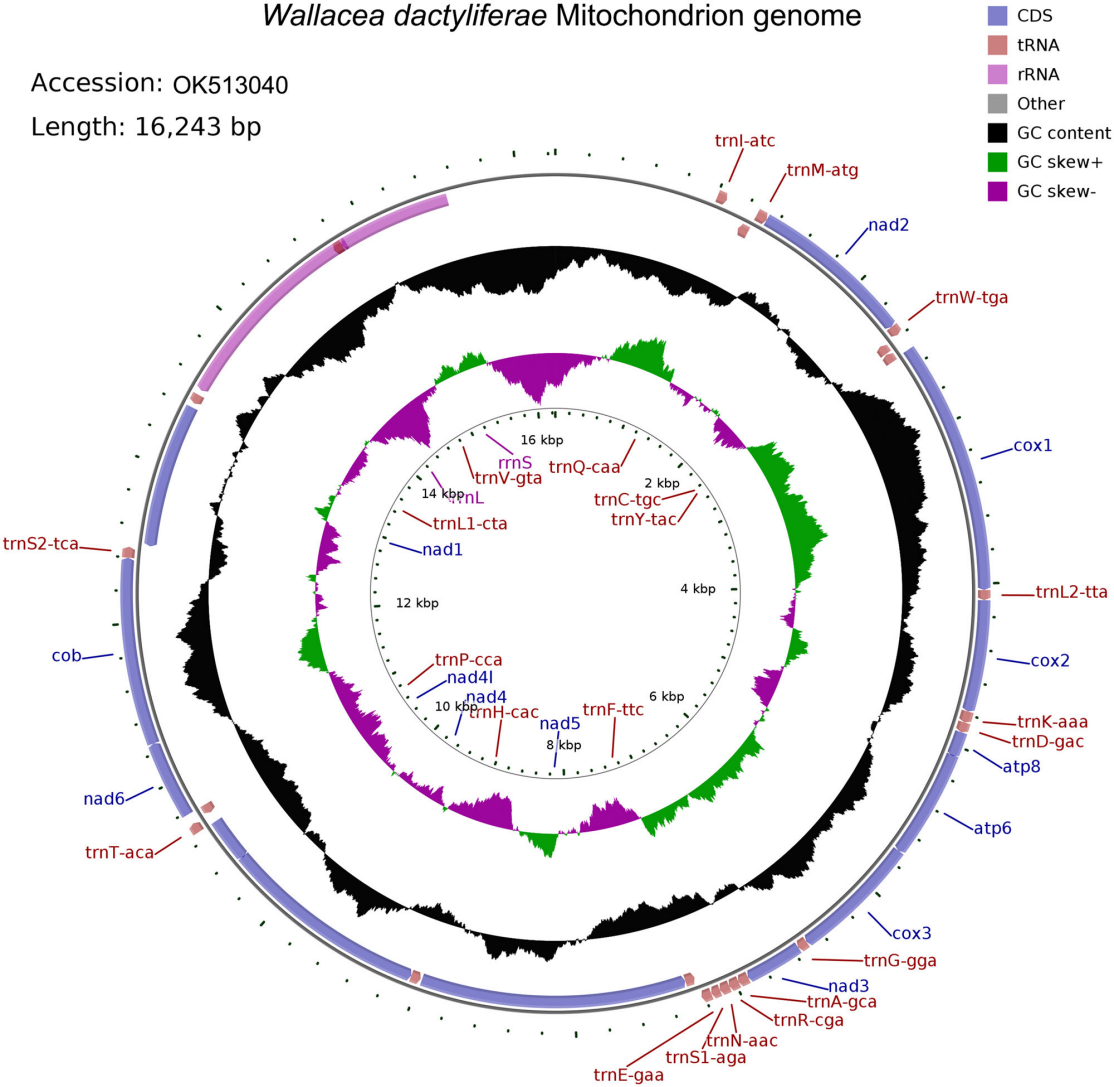
Mitochondrial genome pattern map of *W. dactyliferae*. The assembled mitochondrial genome (16,243 bp) of *W. dactyliferae* (GenBank ID: OK513040) with major features: there are 13 protein-coding genes, 22 tRNA genes, the 12S and 16S rRNA genes; different colors represent different gene blocks, with the arrow pointing in the transcription directions. This map was drawn using the online mitochondrial visualization tool CGView (http://stothard.afns.ualberta.ca/cgview_server/).

The complete mitochondrial genomes of *W. dactyliferae* and other Coleoptera species from Genbank were downloaded and aligned with the MAFFT program (Katoh et al. 2005). The phylogenetic relationship of these species were analyzed by using MEGA 7.0 software based on maximum-likelihood (ML) method with Poisson correction model and 1,000 bootstrap replicates (Tamura et al. 2013). Phylogenetic analysis revealed that species from the same subfamily were clustered together (Figure 3), with *W. dactyliferae* being clustered together with other Cassidinae species. The phylogram is consistent with the taxonomic classification of insects.

**Figure 3.**
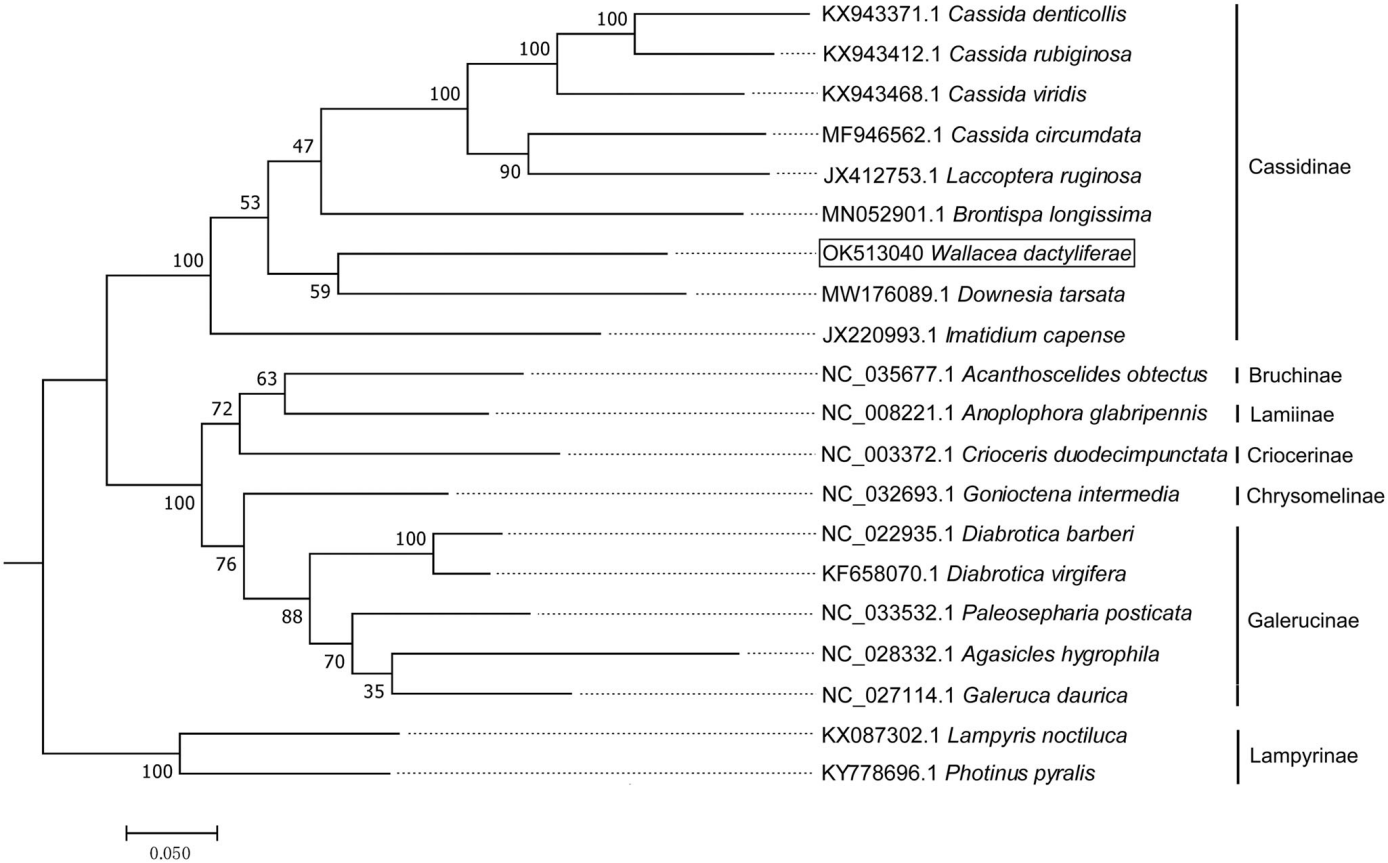
The maximum likelihood (ML) phylogenetic tree of *W. dactyliferae* and other Coleoptera species. Two Lampyrinae species were used as outgroups. Support values next to the nodes are based on 1000 bootstrap replicates.

In a word, this study identifies the entire mitochondrial genome of *W. dactyliferae* for the first time, which can provide necessary DNA molecular data for phylogenetic analyses of the Chrysomelidae.

## Ethical permissions

Insects for which a permit is not required.

## Data Availability

Mitochondrial genome data supporting this study are openly available in GenBank at: https://www.ncbi.nlm.nih.gov/nuccore/OK513040. Associated BioProject, SRA, and BioSample accession numbers are https://www.ncbi.nlm.nih.gov/bioproject/ PRJNA854766, https://www.ncbi.nlm.nih.gov/sra/SRR20706871, and SAMN29446782, respectively.
